# Single-cell landscape and spatial transcriptomic analysis reveals macrophage infiltration and glycolytic metabolism in kidney renal clear cell carcinoma

**DOI:** 10.18632/aging.205128

**Published:** 2023-10-16

**Authors:** Chen-Yueh Wen, Jui-Hu Hsiao, Yen-Dun Tony Tzeng, Renin Chang, Yi-Ling Tsang, Chen-Hsin Kuo, Chia-Jung Li

**Affiliations:** 1 Division of Urology, Department of Surgery, Kaohsiung Veterans General Hospital, Kaohsiung 813, Taiwan; 2 Department of Surgery, Kaohsiung Municipal Minsheng Hospital, Kaohsiung 802, Taiwan; 3 Department of Surgery, Kaohsiung Veterans General Hospital, Kaohsiung 813, Taiwan; 4 Institute of Biomedical Sciences, National Sun Yat-Sen University, Kaohsiung 804, Taiwan; 5 Department of Emergency Medicine, Kaohsiung Veterans General Hospital, Kaohsiung 802, Taiwan; 6 Institute of Physiological Chemistry and Pathobiochemistry and Cells in Motion Interfaculty Centre (CiMIC), University of Münster, Münster 48149, Germany; 7 Genomics Research Center, Academia Sinica, Taipei 115, Taiwan; 8 Department of Obstetrics and Gynecology, Kaohsiung Veterans General Hospital, Kaohsiung 813, Taiwan; 9 Institute of BioPharmaceutical Sciences, National Sun Yat-Sen University, Kaohsiung 804, Taiwan

**Keywords:** PGAM1, glycolytic metabolism, immune infiltration, single cell-RNA sequencing, kidney renal clear cell carcinoma

## Abstract

The present study investigates the clinical relevance of glycolytic factors, specifically PGAM1, in the tumor microenvironment of kidney renal clear cell carcinoma (KIRC). Despite the established role of glycolytic metabolism in cancer pathophysiology, the prognostic implications and key targets in KIRC remain elusive. We analyzed GEO and TCGA datasets to identify DEGs in KIRC and studied their relationship with immune gene expression, survival, tumor stage, gene mutations, and infiltrating immune cells. We explored Pgam1 gene expression in different kidney regions using spatial transcriptomics after mouse kidney injury analysis. Single-cell RNA-sequencing was used to assess the association of PGAM1 with immune cells. Findings were validated with tumor specimens from 60 KIRC patients, correlating PGAM1 expression with clinicopathological features and prognosis using bioinformatics and immunohistochemistry. We demonstrated the expression of central gene regulators in renal cancer in relation to genetic variants, deletions, and tumor microenvironment. Mutations in these hub genes were positively associated with distinct immune cells in six different immune datasets and played a crucial role in immune cell infiltration in KIRC. Single-cell RNA-sequencing revealed that elevated PGAM1 was associated with immune cell infiltration, specifically macrophages. Furthermore, pharmacogenomic analysis of renal cancer cell lines indicated that inactivation of PGAM1 was associated with increased sensitivity to specific small-molecule drugs. Altered PGAM1 in KIRC is associated with disease progression and immune microenvironment. It has diagnostic and prognostic implications, indicating its potential in precision medicine and drug screening.

## INTRODUCTION

Renal cell carcinoma (RCC) represents around 3-5% of all oncological diagnoses worldwide, with higher incidence occurring in Western countries [1]. RCC has several pathological types and the histological classification is important due to different molecular targeted therapy or surgical treatment. Clear cell type accounts for 75% of all RCC cases. It is considered as malignant according to WHO classification and has poor prognosis comparing with other subtypes such as papillary or chromophobe [2, 3]. Although kidney renal clear cell carcinoma (KIRC) is highly aggressive and popular, relatively fewer are known about its etiology. The possible risk factors of KIRC include hereditary diseases or genetic aberration of mTOR pathway proteins [4]. Therefore, it is necessary to consider the newly change in KIRC and to discover novel biomarkers for improving the prognosis of KIRC.

Phosphoglycerate mutase-1 (PGAM1) is a glycolytic enzyme that has been extensively studied in different types of malignancies [5]. Numerous studies have demonstrated that PGAM1 is frequently activated in glycolysis and highly expressed in various types of cancer, including KIRC [6, 7]. In KIRC, PGAM1 has been found to play a crucial role in tumor proliferation, and its high expression is associated with abnormal glycolysis and the formation of KIRC, making it a potential therapeutic target for cancer therapy [8, 9]. In addition to PGAM1, another subfamily of PGAM enzymes, PGAM5, has also been shown to regulate several aspects of cancer cell death, including apoptosis and necrosis, by altering mitochondrial function [10]. The role of PGAM5 in cancer is not as extensively studied as PGAM1, but it is emerging as an important player in cancer cell biology [11]. The dysregulation of PGAM enzymes, particularly PGAM1, plays a significant role in cancer cell metabolism and proliferation, making them attractive targets for cancer therapy. The immune microenvironment has been shown to be crucial for tumorigenesis in KIRC [12]. This study is the first to report the involvement of this metabolic gene in immune cell infiltration in KIRC. Further research is needed to fully understand the mechanisms underlying their involvement in cancer progression and to develop effective therapeutic strategies.

The aim of our study was to explore the theragnostic potential of PGAM1 in KIRC through an integrated analysis. This included analyzing differential gene expression, protein correlation, pathway analysis, and prognostic analysis across various tumor types and stages. We also examined the correlation of PGAM1 expression with immune-infiltrating cells and immunomodulatory factors. Our findings indicate that PGAM1 could serve as a strong prognostic biomarker and is closely associated with immune mechanisms, highlighting its potential as an immunotherapeutic agent for KIRC.

## MATERIALS AND METHODS

### Analysis of gene expression differences and prognostic significance in KIRC

In this study on differential expression of KIRC genes and their prognostic significance, various databases and tools were used to analyze gene expression levels in KIRC tumors and adjacent normal tissues, and survival analysis was performed as described in a previous study [13, 14]. Briefly, TNMplot, UALCAN (The University of ALabama at Birmingham CANcer data analysis Portal), and Gene Expression Omnibus (GEO) were accessed to retrieve relevant data. The Human Protein Atlas was also consulted to obtain immunohistochemical (IHC) staining images for further analysis. In conducting survival analysis, the Kaplan-Meier plotter tool was utilized to assess the relationship between clinical stages of KIRC and various factors such as immune cell content and tumor mutational burdens. To determine optimal patient groupings for survival analysis, the tool was configured to use the “Auto select best cut off” feature, which selects the best-performing cutoff value. Overall, the various analyses performed in this study provide important insights into the gene expression and prognostic significance of KIRC, and may inform future research and clinical decision-making in this area.

### Single-cell transcriptomic and immune profiling analysis

Single-cell RNA sequencing (scRNA-seq) has emerged as a powerful tool to explore cellular heterogeneity and gene expression at the single-cell level. In this study, scRNA-seq data were obtained from the GSE159115 and GSE121636 datasets of the GEO database. Quality control (QC) was performed using the R package Seurat to ensure the inclusion of high-quality cells and reduce the influence of batch effects. To identify distinct cell subpopulations, uniform manifold approximation and projection (UMAP) clustering was performed using the “BiocManager” and Gene Set Variation Analysis (“GSVA”) packages in R. Cell type annotation was performed by comparing the expression profiles of previously recognized cellular marker genes with those of the identified cell subpopulations using the “SingleR” package in R. The association between gene expression and immune cell infiltration/abundance was explored using the “Gene” module of TIMER, focusing on macrophages, dendritic cells (DC), and CD4+ T, CD8+ T, and B cells. Finally, the association between immune infiltration and overall survival of KIRC patients was estimated using several algorithms, including TIMER, EPIC, MCPCOUNTER, CIBERSORT, CIBERSORT-ABS, QUANTISEQ, and XCELL.

### Processing of spatial transcriptomics data using seurat algorithm

We performed analysis based on previously published articles and established spatial transcriptome data of kidney injury model animals [15]. The spatial transcriptomics data were processed using the R package Seurat (v4.1.0) and log normalization was performed to normalize the data. To integrate Seurat objects into a single ST dataset and remove batch effects, we utilized the functions SelectIntegrationFeatures, FindIntegrationAnchors, and IntegrateData. To reduce dimensionality, we performed the function RunPCA, and then used the functions FindNeighbors and FindClusters to cluster similar ST points. Initially, different clusters were divided based on hematoxylin-eosin stained (H&E) sections and annotated by unsupervised cluster analysis. Upon annotating clusters with cell markers, we observed that some clusters highly expressed multiple cell markers. To address this, we employed the ssGSEA algorithm to score common cell types based on the average expression matrix of different clusters, which was confirmed to be more effective in ST [16].

### Analysis of human KIRC specimens

Tissue microarray (TMA) slides (CL2) consisting of human renal cancer, metastatic, and normal tissues were procured from SuperBioChips Laboratories (Seoul, Republic of Korea). To perform an immunohistochemistry (IHC), the protocol described in a prior study [17] was followed.

### Colocalization analysis of immunofluorescence staining

We employed a method that has been described previously, which is summarized as follows [18]. Immunofluorescence analysis was performed by fixing the tissue with 4% paraformaldehyde at room temperature for 10 minutes, permeabilizing it with 0.2% Triton X-100 for 5 minutes, blocking it with 5% BSA for 30 minutes, and incubating it overnight at 4° C with the specified primary antibodies PGAM1_A4170 and CD163_A22619 purchased from Abclonal (Woburn, MA, USA). After washing with PBST, the samples were incubated with secondary antibodies for 30 minutes at room temperature. The BX61VS® Fully Motorized Fluorescence Microscope (Olympus Corporation, Tokyo, Japan) at ×20 magnification was used to digitize all glass slides with high precision. The whole-slide images were viewed and analyzed with Olympus VS-ASW® software at Li-Tzung Pathology Laboratory (Kaohsiung, Taiwan).

### Pharmacogenetic prediction model

We developed a pharmacogenetic prediction model to analyze drug sensitivity based on PGAM1 expression, using the knockdown-screen data repository of the Genomics of Drug Sensitivity in Cancer (GDSC) algorithm in Q-omics v.1.0. Pearson correlation coefficient analysis was performed to assess the correlation between PGAM1 expression levels and drug dose levels [19].

### Statistical analysis

The statistical analysis in this study followed the methods described in a previous publication [20]. Pearson’s correlation coefficient was used to evaluate gene expression correlation, while a t-test or Fisher’s exact test was used for the comparison between two groups, and one-way ANOVA was used for comparison within one group, all analyzed using GraphPad Prism software (GraphPad Software, La Jolla, CA, USA). A p-value of less than 0.05 was considered statistically significant.

## RESULTS

### Differential expression and genetic variation analysis of PGAM1 in KIRC

**Figure 1:**
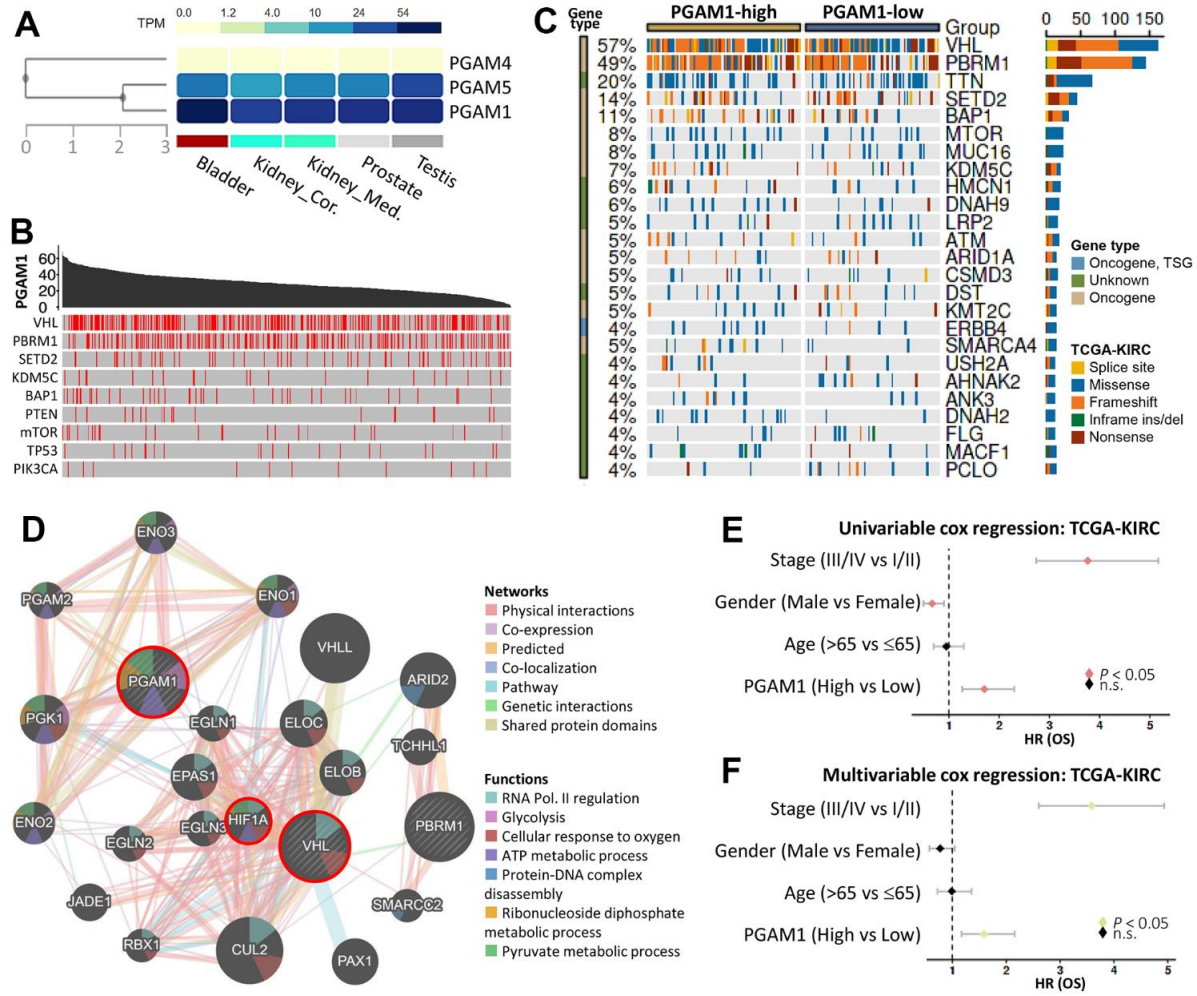
**Gene landscape and characteristics of PGAM1 in KIRC.** (**A**) PGAM1 gene expression levels in the male urinary system were examined. (**B**) The relationship between PGAM1 and nine highly mutated genes in KIRC was investigated, with mutation sites indicated by red lines. (**C**) The frequency of mutations was compared between PGAM1-high and PGAM1-low groups using Fisher’s exact test. Mutation types, driver mutation types, and groups are shown in the right panel. (**D**) A PGAM1 interaction network was generated using the Reactome database. (**E**, **F**) Univariate and multivariate Cox regression models were used to calculate hazard ratios for PGAM1 at different stages of KIRC.

We first conducted an analysis of the expression levels of PGAM family genes in the male reproductive system and observed that PGAM1 exhibited higher expression in male reproductive organs of fat fruits (Figure 1A). We subsequently explored the association of PGAM1 mutations with common cancer progression genes, such as VHL, PBRM1, and SETD2 (Figure 1B). Subsequently, we conducted an analysis in which we extracted data pertaining to PGAM1 expression from the TCGA database, focusing on patients diagnosed with Kidney Renal Clear Cell Carcinoma (KIRC). This information was presented using a waterfall plot, offering a visual representation of the top 25 genes that exhibited notable alterations in response to PGAM1 expression changes. This analysis delved into the genetic variations that occur at different levels of PGAM1 expression and their connections with genes that are frequently found to be mutated in KIRC. The insights gained from this examination are depicted graphically in Figure 1C, providing a comprehensive view of these associations and their potential implications in the context of KIRC. The mutation landscape identified various types of mutations, including splice site, missense, frameshift, and nonsense mutations, as well as in-frame ins/dels. Moreover, we used protein-protein interactions to evaluate regulatory network maps and inferred underlying mechanisms. Our analysis revealed that PGAM1 was associated with VHL, which was consistent with the results from Figure 1C. Furthermore, we found that the intermediate factor connecting these two genes was HIF1A (Figure 1D). We conducted univariate and multivariate Cox regression analyses to evaluate the association of PGAM1 expression with OS. Our findings indicated that stage, sex, age, and PGAM1 (high vs low) were significantly associated with OS (Figure 1E). Finally, we applied multivariate Cox regression analysis to the same variables and found that the risk score could serve as an independent prognostic factor (P < 0.05) (Figure 1F).

**Figure 2:**
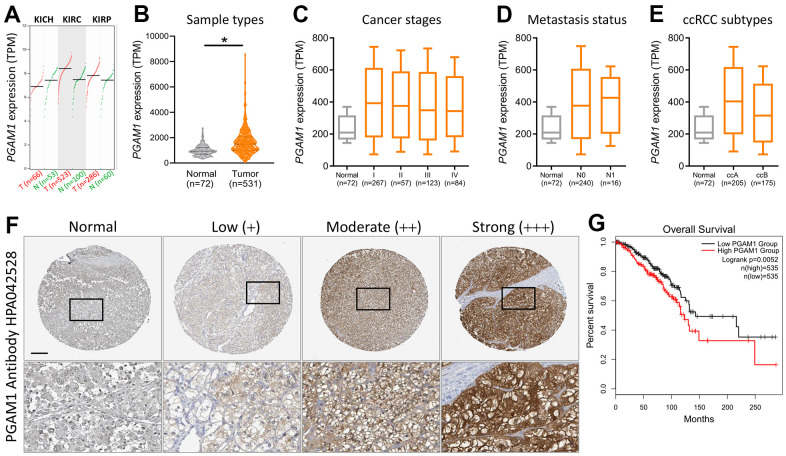
**Evaluation of the diagnostic potential of PGAM1 expression in KIRC biopsy specimens.** (**A**) PGAM1 gene expression levels in renal cancer. (**B**) Comparison of PGAM1 expression between KIRC tumor and non-tumor tissues. Boxplots depicting PGAM1 expression levels across different stages (**C**), metastasis status (**D**), and ccRCC subtypes (**E**) of KIRC. (**F**) Comparative immunohistochemical analysis of PGAM1 expression in KIRC tissue samples from four different patients based on the Human Protein Atlas. (**G**) Prognostic significance of PGAM1 mRNA levels for overall survival, as determined using the Kaplan-Meier plotter dataset.

Subsequently, we conducted a comprehensive assessment of various kidney cancers and discovered that the levels of phosphoglycerate mutase-1 (PGAM1) were elevated in both clear cell renal cell carcinoma (KIRC) and papillary renal cell carcinoma (KIRP) but reduced in chromophobe renal cell carcinoma (KICH) (Figure 2A). Upon scrutinizing the expression of PGAM1 across diverse sample types, cancer stages, metastasis stages, and ccRCC subtypes in The Cancer Genome Atlas (TCGA) (Figure 2B–2E), we observed that PGAM1 expression was significantly amplified in the first stage of metastatic KIRC, and both subtypes of ccRCC exhibited an upward trend. Furthermore, analysis of immunohistochemical staining data from the Human Protein Atlas verified that the PGAM1 protein was upregulated in KIRC tumor tissues relative to normal tissues (Figure 2F). Strikingly, Kaplan-Meier analysis revealed that patients with elevated PGAM1 immunohistochemical scores had a shorter overall survival time (Figure 2G), underscoring the potential clinical significance of PGAM1 in ccRCC.

### Spatial transcriptomics evaluation of PGAM1 expression in injured kidneys

**Figure 3:**
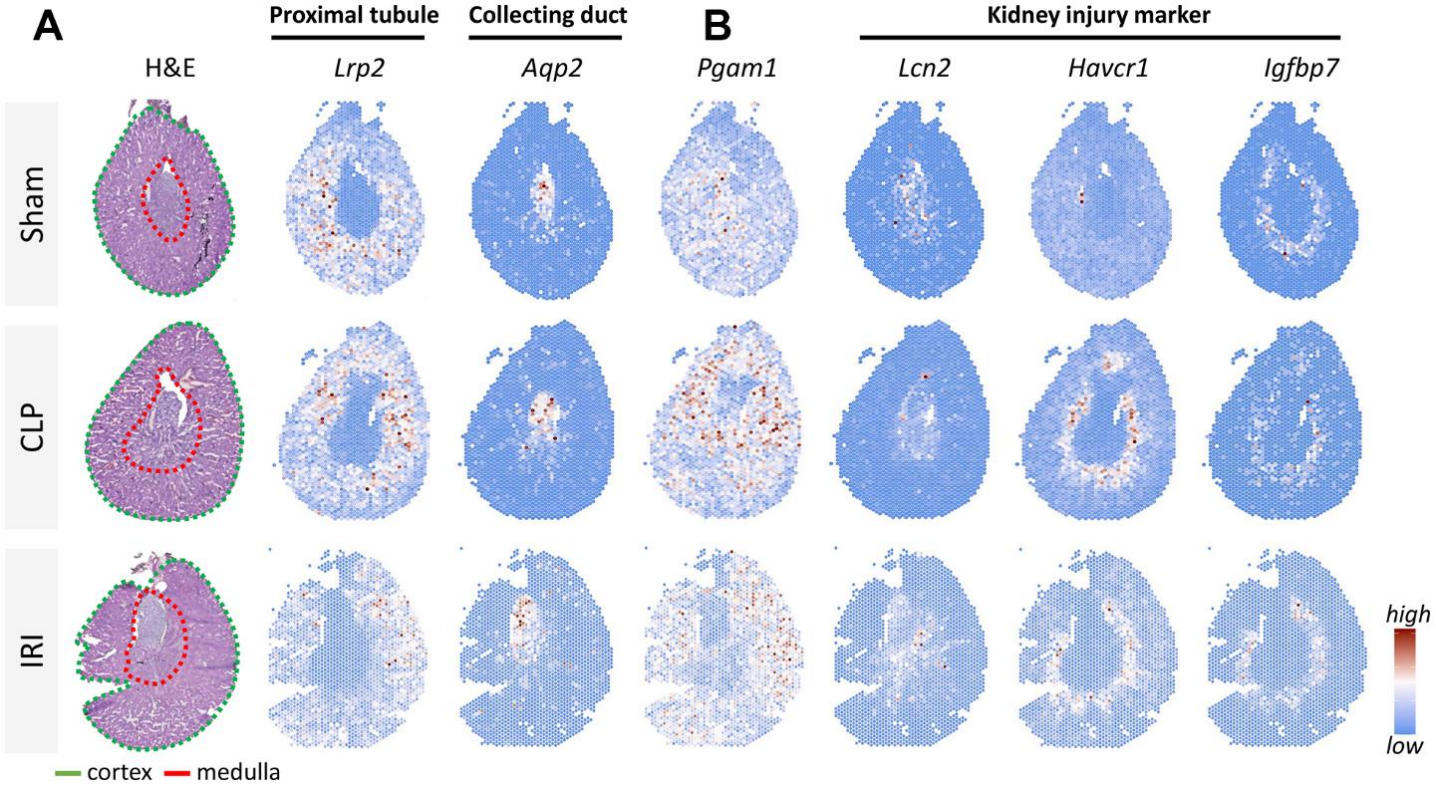
**Resolving spatial relationships of cell type and gene expression using spatial transcriptomics in a mouse kidney injury model.** (**A**) H&E-stained sections of 3 mouse models: sham operation, ischemia/reperfusion injury (IRI), and cecal ligation and puncture (CLP), respectively. Different regions of the cortex (Lrp2) and medulla (Aqp2) were labeled using tissue-specific biomarkers. (**B**) Analysis of Pgam1 and different biomarkers of renal injury (Lcn2, Kim1, Havcr1).

To assess the changes and distribution of PGAM1 expression in renal injury, we conducted an analysis of previously published data [15] that utilized two models of injured kidneys for spatial transcriptomic sequencing. The intact kidney tissue was sectioned coronally to expose each major physiologic region, cryosectioned and placed on a tissue capture area on a dedicated 10× Genomics Visium slide embedded with oligonucleotide sequences. Marker gene expression was visualized using Seurat, and distinct regions were identified by biomarkers in proximal tubules (Lrp2) and collecting ducts (Aqp2) (Figure 3A). Our results showed that PGAM1 expression was higher in the proximal tubules of renal injury compared to the control group. Furthermore, we also analyzed the gene levels of common kidney injury biomarkers as controls, including lipocalin/lipocalin 2 (Lcn2), kidney injury molecule 1 (Havcr1), insulin-like growth factor binding protein 7 (Igfbp7), all of which have significantly high expression in kidney injury. Therefore, we concluded that the metabolic gene PGAM1 was highly expressed in the damaged kidney, which may be related to the damage of this region and the change of metabolic pathway.

### Correlation analysis between PGAM1 and infiltrating immune cells

**Figure 4:**
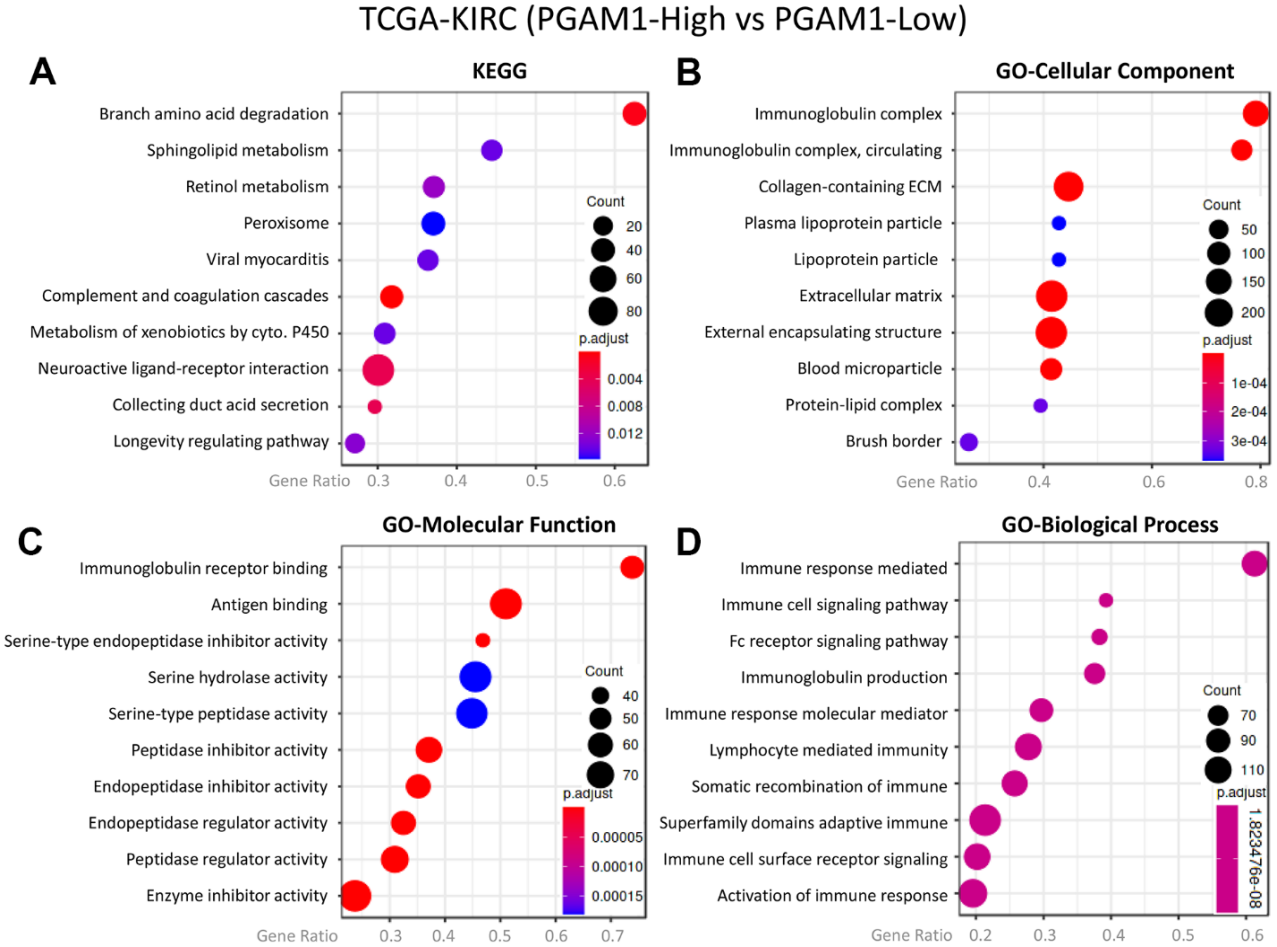
**The co-expression genes of PGAM1 in KIRC were subjected to enrichment analysis.** The target genes were analyzed using (**A**) Kyoto Encyclopedia of Genes and Genomes (KEGG) pathway, (**B**) cellular component, focusing on (**C**) molecular function and (**D**) biological process.

In light of the role of the tumor immune microenvironment (TIME) in promoting kidney damage and recruiting immune cells, we further investigated the relationship between metabolism and the immune response in tumors. Our analysis showed that overexpression of PGAM1 resulted in Branch amino acid degradation, as reflected in the KEGG pathway analysis (Figure 4A). Moreover, the activation of the Immunoglobulin complex was observed in the GO-cellular component analysis (Figure 4B), and the same result was reflected in the GO-molecular function analysis, where the ratio of Immunoglobulin receptor binding and Immune response-mediated biological function was greatly increased (Figure 4C, 4D). Based on these findings, we hypothesize that kidney damage, which requires high energy, not only leads to metabolic conversion and abnormality but also promotes infiltration of immune cells, playing a significant role in the tumor immune microenvironment. The activation of the Immunoglobulin complex and increased Immunoglobulin receptor binding observed in our analysis suggests that immune cells may be recruited to the damaged kidney, contributing to the immune response in tumors.

### Evaluation of PGAM1 transcriptome using single-cell RNA sequencing database analysis

**Figure 5:**
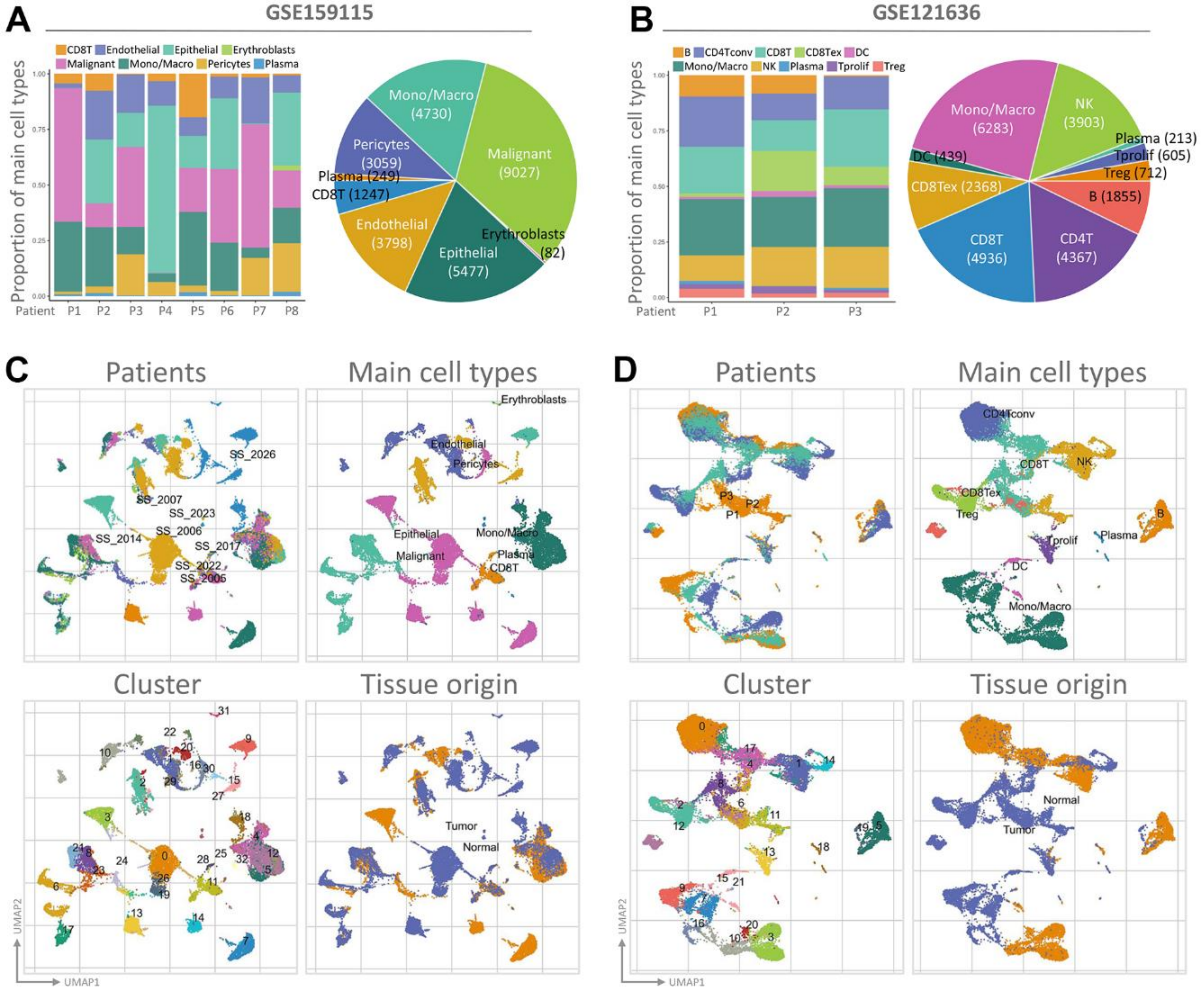
**The use of single-cell RNA sequencing analysis has allowed for the identification of immune cell populations.** (**A**, **B**) The relative proportions of each cell type found within the two datasets, while showcasing the proportion of integrated immune cells present within the databases. UMAP is an abbreviation for the Unified Manifold Approximation and Projection technique used in this study. Visual representations of all KIRC cells from both GEO datasets are depicted in (**C**, **D**) through the utilization of Unified Manifold Approximation and Projection (UMAP) and assigned specific colors according to clusters.

**Figure 6:**
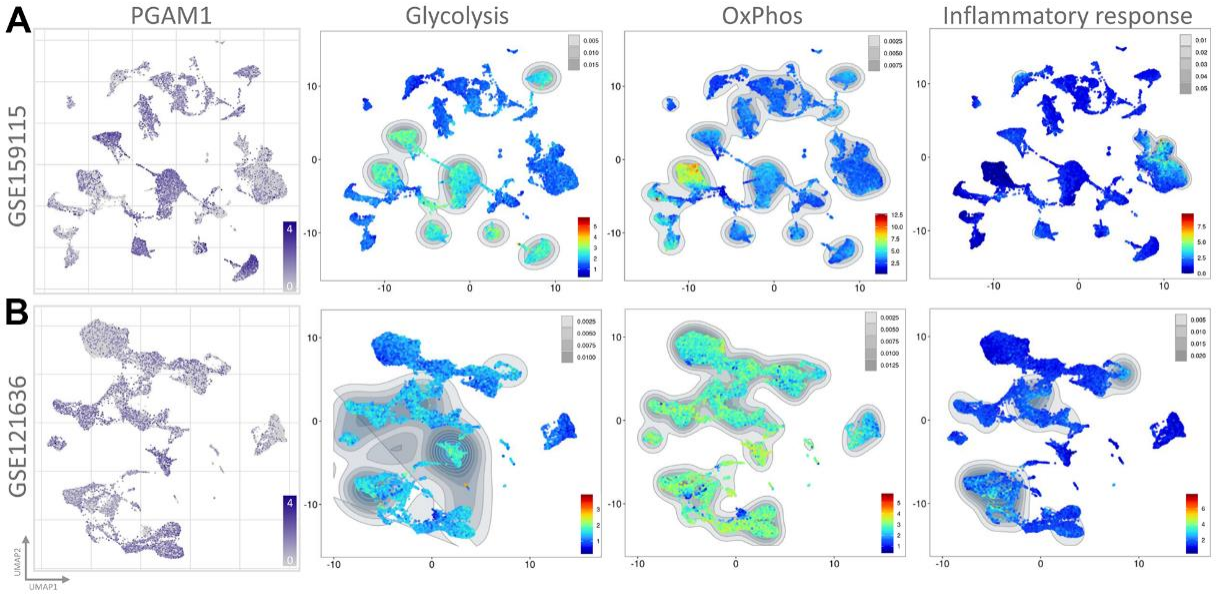
**The single-cell transcriptomes of patient-derived cultures treated with PGAM1 were presented.** The expression clusters of PGAM1 were visualized using UMAP plots in (**A**, **B**), while the UMAP plots of each distinct cluster were analyzed through Gene Set Enrichment Analysis (GSEA).

To assess the transcriptome of PGAM1 in renal cancer at the single-cell level and to explore the heterogeneity of different cell types in the renal cancer microenvironment, we conducted an analysis of two publicly available single-cell RNA-sequencing databases (GSE159115 and GSE121636). A total of 8 samples from GSE159115, comprising 8 types of cells and 32 clusters, and 3 samples from GSE121636, comprising 10 cells and 21 clusters, were included in the study. Using UMAP plots, we identified and visualized 8 and 10 major cell populations in GSE159115 and GSE121636, respectively (Figure 5A, 5B). After quality control and batch effect removal, we analyzed 27,669 (GSE159115) and 25,681 (GSE121636) cells, respectively. We determined cell type-specific markers for each cluster based on the top-ranked differentially expressed genes, which were used for cell type classification. Figure 5C, 5D shows the clinical information for each cell population, where differences in the corresponding proportions of each cluster were observed for different clinical characteristics. We further analyzed the expression and distribution of PGAM1 in these two single-cell sequencing databases. As shown in Figure 6A, 6B, we found that PGAM1 expression levels in different cell types of renal cancer were high in the same region as the high glycolysis model. In addition, oxidative phosphorylation in Figure 6B was generally increased, which was also reflected in the Inflammatory response.

### PGAM1 over-expression is correlated with immune infiltration

**Figure 7:**
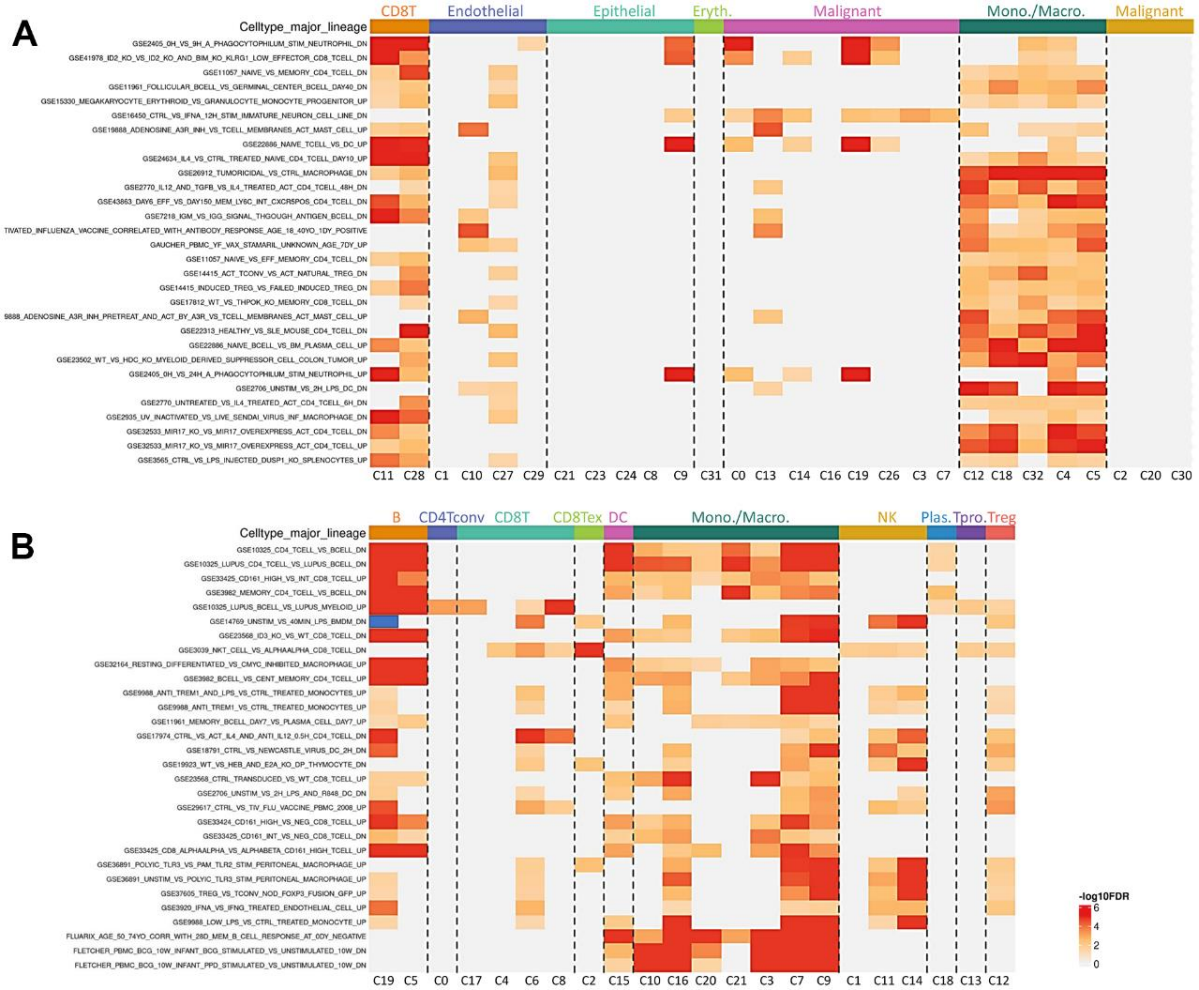
**The impact of PGAM1 on the Tumor Immune MicroEnvironment (TIME) was investigated.** Immunological analyses of immune infiltrates and immunosuppressants were carried out using GSE159115 and GSE121636 databases, respectively, as shown in (**A**). Furthermore, a heatmap was presented to depict the correlation between PGAM1 expression and lymphocytic infiltration in human cancers (**B**).

To investigate the potential relationship between KIRC and PGAM1, we performed gene set enrichment analysis (GSEA) using differentially expressed genes (DEGs) and identified highly correlated clusters with macrophages in two single-cell RNA sequencing databases (Figure 7A, 7B). However, macrophages exhibit tissue residency and possess pro- or anti-inflammatory functions. Given our previous findings of PGAM1 overexpression in renal injury models and human KIRC, and the crucial role of the tumor immune microenvironment (TIME) in tumor growth, metastasis, and immune evasion, we further analyzed the relationship between PGAM1 and tumor-immune interactions.

**Figure 8:**
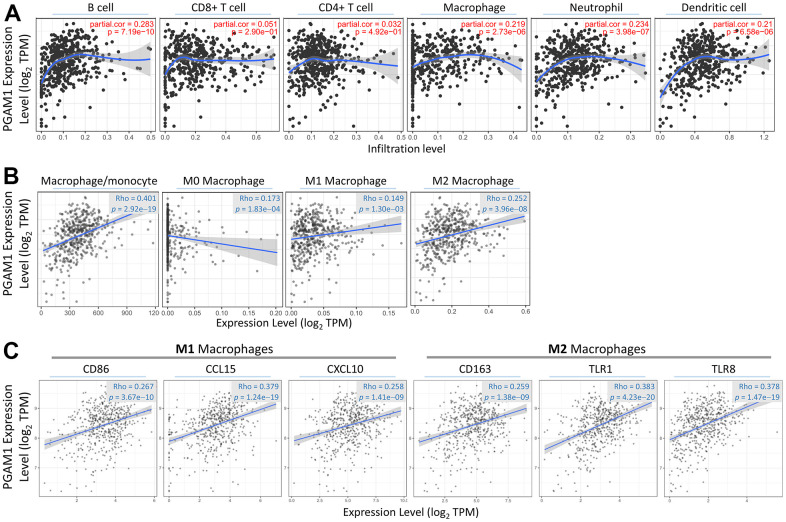
**Investigating the link between PGAM1 and immunization.** (**A**) TIMER analysis determines the correlation between PGAM1 expression and six different immune cells in KIRC. (**B**) The correlation between PGAM1 levels and various types of macrophages is assessed. (**C**) Additionally, the correlation between PGAM1 and genes related to macrophages is investigated.

Based on the hypothesis that PGAM1 expression might be associated with the tumor microenvironment of renal cancer and differ significantly in macrophages (Figure 8A), we assessed the correlation of macrophage subtypes (M0, M1, and M2) with PGAM1. Our analysis showed a positive correlation between PGAM1 and M1 and M2, while M0 showed the opposite trend (Figure 8B). We further analyzed macrophage biomarkers and found that PGAM1 was positively correlated with CD86, CCL15, CXCL10, CD163, TLR1, and TLR8 (Figure 8C). This result indicates that patients with high PGAM1 expression exhibited elevated renal cell score, immune score, and ESTIMATE score.

**Figure 9:**
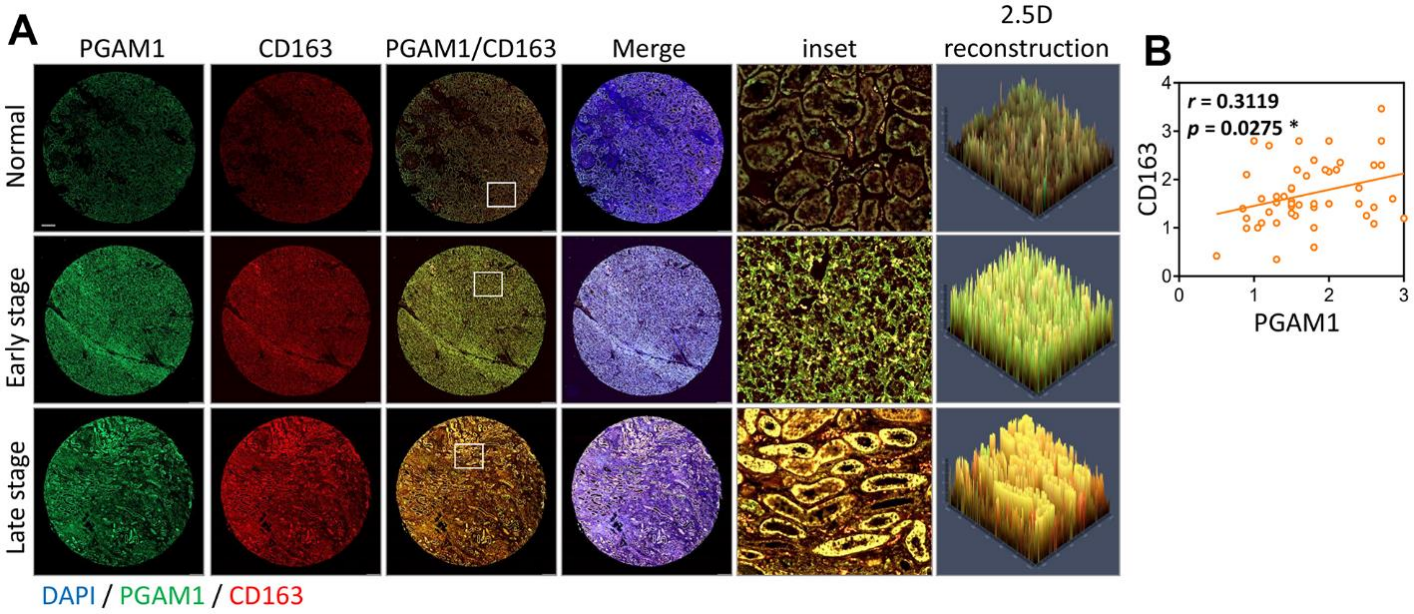
**Analyzing the co-occurrence of PGAM1 and CD163 in tumor biopsies during KIRC development.** (**A**) Merge indicates PGAM1/CD163/DAPI; inset indicates local magnification; 2.5D reconstructed image shows the local fluorescence changes, morphology and fluorescence intensity. The merge image displays PGAM1/CD163/DAPI, while the inset exhibits a local magnification. The 2.5D reconstructed image showcases local fluorescence changes, morphology, and fluorescence intensity. (**B**) The Pearson's correlation coefficient was employed to visualize the degree of overlap between PGAM1 and CD163 fluorescence signals.

To validate the connection between PGAM1 and CD163 in KIRC, we investigated the co-localization of these two proteins in TMA through triple-labeling the whole KIRC with immunofluorescence for PGAM1, CD163, and DAPI. We segmented the human KIRC TMAs into early and late stages and analyzed their fluorescence intensity through panoramic tissue scanning. In Figure 9A, the red fluorescent PGAM1 and green fluorescent CD163 demonstrated that the normal group had a higher intensity than the tumor group, and the red fluorescent intensity was higher in the early stage than in the late stage, which was similar to Figure 2F. The green fluorescence was consistent with the late stage of the normal group being lower than that of the tumor group. The 2.5D image reconstruction showed a clear overlapping phenomenon of PGAM1 and CD163 signals. After quantitative analysis by fluorescence, we confirmed that PGAM1 was positively correlated with CD163 (r = 0.3119; p = 0.0275) (Figure 9B). Taken together, these findings indicate that PGAM1 overexpression is associated with the active TIME of macrophages.

### Pharmacogenetic screening for potential PGAM1 inhibitors in KIRC

**Figure 10:**
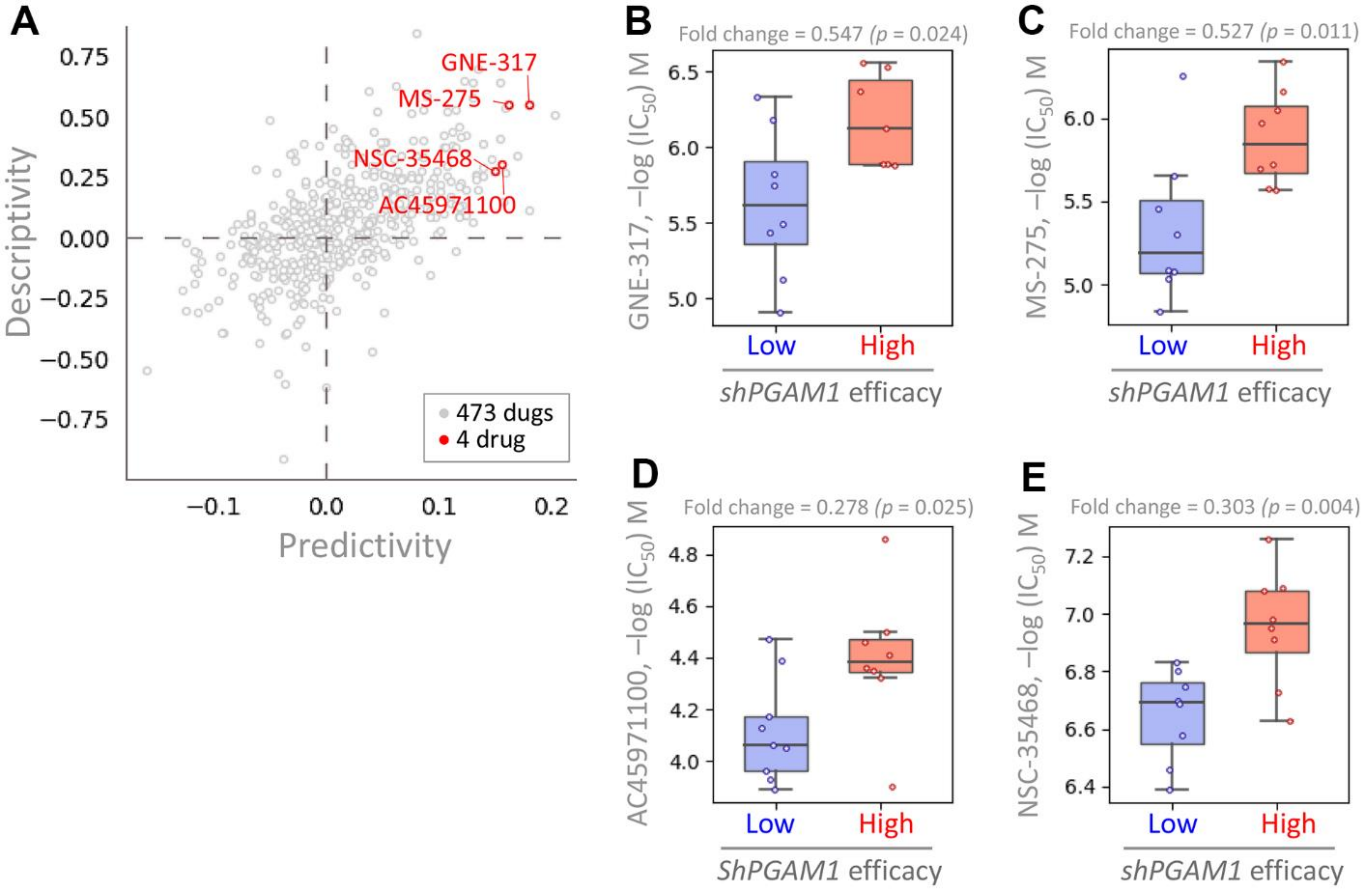
**Analysis of drug sensitivity and cytotoxicity in renal cancer cells.** (**A**) The PGAM1 gene was queried in the pharmacogenetics database to identify gene signatures and potential drugs. Predictivity refers to the fold change in efficacy of short hairpin PGAM1 (shPGAM1), which indicates the efficiency of PGAM1 knockdown using shRNA, between cells with high and low response to the target drug. The drug sensitivity of the shPGAM1 gene to various chemical drugs was evaluated in KIRC cell lines. The boxplots show the log of the half maximal inhibitory concentration (IC_50_) values of GNE-317 (**B**), MS-275 (**C**), AC45971100 (**D**), and NSC-35468 (**E**).

In this study, we aimed to identify drugs with potential efficacy against KIRC by analyzing the GDSC repository for drugs that exhibit enhanced potency in the presence of high PGAM1 expression. We performed a cross-correlation analysis to investigate the effects of 473 drugs on shRNA-mediated PGAM1 in various KIRC cells. Our analysis identified four drugs, namely GNE-317, MS-275, AC45971100, and NSC-35468, that displayed altered potency (Figure 10A). Notably, KIRC cell lines with high shPGAM1 efficiency exhibited increased sensitivity to these drugs (Figure 10B–10E). Collectively, these findings suggest that GNE-317, MS-275, AC45971100, and NSC-35468 hold potential as anticancer agents targeting PGAM1 to regulate KIRC growth.

## DISCUSSION

Kidney injury and KIRC are two distinct conditions that can be associated with metabolic and glycolysis changes [21]. Chronic kidney disease (CKD) refers to a progressive loss of kidney function over time [22]. This can be caused by a variety of factors, including high blood pressure, diabetes, and other underlying medical conditions. In many cases, CKD is asymptomatic and may not be diagnosed until it has reached an advanced stage [23]. KIRC, on the other hand, is a type of kidney cancer that arises from the cells lining the small tubes within the kidney. This type of cancer is known for its potential to spread quickly to other parts of the body and can be difficult to treat [24]. Both CKD and KIRC have been associated with changes in metabolism and glycolysis. Metabolism refers to the chemical processes that occur within cells to generate energy, while glycolysis is the breakdown of glucose into energy [25, 26].

In cases of CKD, changes in metabolism and glycolysis may be related to the impaired function of the kidney. Studies have shown that in CKD patients, there is a decrease in the activity of key enzymes involved in glycolysis, such as hexokinase and pyruvate kinase [22]. This leads to a reduced ability of the kidneys to generate energy from glucose and may contribute to the development of insulin resistance and other metabolic abnormalities [27, 28]. Furthermore, CKD is associated with alterations in the metabolism of lipids and proteins, which can lead to the accumulation of toxic byproducts that can damage the kidneys further. The metabolic changes associated with CKD can have significant impacts on the function of the kidney [29].

One of the key metabolic alterations in KIRC is an increase in glycolysis, which is a process by which glucose is broken down to produce energy. This increase in glycolysis is thought to be due to mutations in the von Hippel-Lindau (VHL) tumor suppressor gene, which is commonly found in KIRC. The loss of VHL function leads to the stabilization of hypoxia-inducible factor (HIF), which in turn promotes glycolysis and angiogenesis [30, 31]. In addition to glycolysis, KIRC is also associated with changes in other metabolic pathways. For example, there is evidence to suggest that the tumor cells may undergo a shift towards increased fatty acid oxidation, which can provide an alternative source of energy for the tumor cells. There are also changes in the tricarboxylic acid (TCA) cycle, which is a key metabolic pathway that generates energy in the form of ATP [32, 33]. Understanding the metabolic and glycolysis changes associated with CKD and KIRC is an important area of research that may lead to new diagnostic and treatment approaches for these conditions.

The PGAM1 gene encodes a mutase that facilitates the reversible conversion of 3-phosphoglycerate (3-PGA) to 2-phosphoglycerate (2-PGA) in the glycolytic pathway, and its emerging research focus is on the regulation of cell damage and energy metabolism [34]. However, the clinical relevance, association with TIME, and therapeutic significance of PGAM1 in KIRC patients remain unknown. In this study, we employed a multi-omics approach, experimental studies, and clinical validation to uncover novel roles of PGAM1 in KIRC. Our findings demonstrate that elevated PGAM1 expression is an independent diagnostic biomarker that correlates with advanced clinical status and poor prognosis in KIRC. Moreover, we identified macrophages as key contributors to PGAM1 upregulation in tumor tissues, and analysis of spatial transcriptome and single-cell sequencing showed that PGAM1 expression is associated with immune TIME [35]. Mechanistically, PGAM1 overexpression in KIRC may be attributed to metabolic abnormalities or the high energy requirements of metastatic cancer cells, as PGAM1 and HIF1A exhibit direct regulation [11]. Overall, our study highlights the intricate relationship between metabolism and the immune response in the tumor microenvironment and provides insight into the potential mechanisms underlying kidney damage and the recruitment of immune cells.

In our pharmacogenomic data, we present four potential small-molecule drugs. GNE-317 is a PI3K/mTOR inhibitor, and our Figure 1B indicates an association between PGAM1 mutations and PIK3CA/mTOR alterations. Previous studies have shown that blocking PI3K/mTOR significantly inhibits various cancers, including gastric cancer, colorectal cancer, cervical cancer, and more [36–38]. GNE-275 is an HDAC inhibitor widely used in the treatment of various cancers, including prostate cancer, Diffuse Intrinsic Pontine Gliomas, and liver cancer [39–41]. AC45971100, also known as fluometuron, is an immune modulator that may exert its effects by inhibiting mitochondrial enzyme dihydroorotate dehydrogenase and effectively suppressing the growth of breast cancer cells [42]. NSC35468, or Podophyllotoxin bromide, is less commonly reported but has been suggested in literature to induce mitochondrial inner membrane depolarization and caspase-dependent apoptosis in colorectal cancer [43].

## CONCLUSIONS

**Figure 11:**
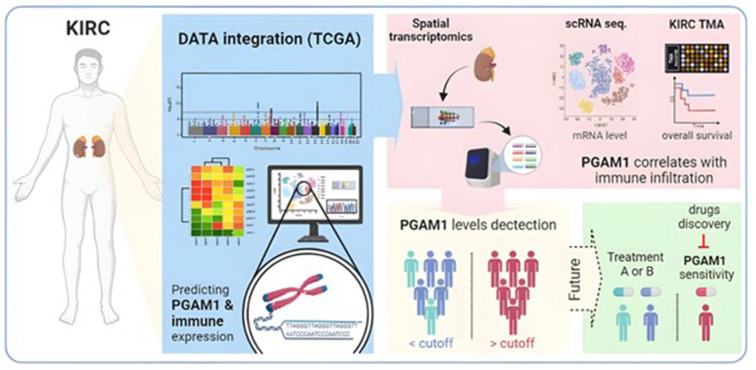
The proposed model depicts the potential significance of PGAM1 in various aspects of KIRC, such as diagnosis, prognosis, tumor immune microenvironment, and potential precision treatments.

The function of the PGAM1 gene in renal cancer remains unknown. Our study demonstrates that increased PGAM1 expression is linked to poor prognosis in KIRC and may impact the tumor microenvironment and macrophage infiltration. As a result, PGAM1 could be a valuable diagnostic, prognostic, and immune-related therapeutic target for KIRC (Figure 11). Additional research is required to confirm our findings and investigate the immunomodulatory effects and mechanisms of PGAM1 in KIRC. This study highlights the inverse relationship between PGAM1 and immune response and identifies several genes worthy of further study as potential diagnostic biomarkers, therapeutic targets, and treatment options.
